# *In vitro* kinetics and inhibition of krait snake’s venom acetylcholinesterase by *Calligonum polygonoides* extract in relation to the treatment of Alzheimer’s disease

**DOI:** 10.22038/IJBMS.2018.28884.6979

**Published:** 2018-08

**Authors:** Mushtaq Ahmed, Aden Razaq, Abdul Razaq, Nadia Mushtaq, Rahmat Ali Khan

**Affiliations:** 1 Department of Biotechnology, Faculty of Biological Sciences, University of Science & Technology, Bannu 28100, KPK-Pakistan; 2 Bannu Medical College Bannu, KPK, Pakistan

**Keywords:** Aging, *Calligonum polygonoides*, Dementia, Ellman assay, Neurodegeneration, Venom protein

## Abstract

**Objective(s):**

The objective of this study was to evaluate pharmacological effect of *Calligonum polygonoides *against Krait snake’s venom acetylcholinesterase (AChE) and to extent it for the treatment of Alzheimer’s disease.

**Materials and Methods:**

Acetylcholinesterase activity was measured using Ellman method with some modification. The kinetic studies of methanolic extract of *C. polygonoides* against krait (*Bungarus Sindanus*) snake venom AChE was measured with the help of the Lineweaver Burk double reciprocal plot.

**Results:**

Statistical data of the results showed that *C. polygonoides* extract inhibited the krait venom AChE in concentration dependent manner. Kinetic analysis using Line weaver Burk plot revealed that *C. polygonoides* caused mixed type of inhibition i.e. km value increased (25-106.6%) while Vmax decreased from 15 to 50% with an increase of *C. polygonoides* extract concentrations (100-300 µg/ml). The calculated IC_50_ value of *C. polygonoides* was found to be 250 µg/ml.

**Conclusion:**

*C. polygonoides* extract can be considered as a therapeutic agent to cure Alzheimer’s disease via inhibition of AChE activity to increase the level of acetylcholine in the body system.

## Introduction

In developing countries, demises from snake bite are somewhat prodigious and remain an ignored issue (1). The importance of snake bite is more for the inhabitants of these countries and mostly for those who works in agricultural fields. Snakes alone are estimated to perpetrate 2.5 million venomous bites each year, resulting in about 125,000 deaths. The actual number may be much larger. Southeast Asia, Pakistan, India, Brazil, and areas of Africa have the most deaths due to snake bite (2). Anti-venoms are the only effective way of treating the sufferers, but they do not have equal access to anti-venoms. For the reason aforesaid, researcher’s interest to find out easy, accessible and inexpensive source to alleviate the condition resulting from the snake’s bite has evidently augmented. An interesting solution is to practice plants extract, fraction or purified compounds to help out the victimized population (3-7). In Asia, most of the deaths are reported due to snake’s bite especially snakes of the members of Elapidae family. Family Elapidae venom contain large amount of acetylcholinesterase (AChE), which causes the inactivation of acetylcholine (physiological events controller) by the enzymatic interruption (8, 9).

Previously, it has been reported that plant extracts possess potent snake venom neutralizing capacity for *Vipera russellii* and *Naja kaouthia* venom (10). Plants having AChE inhibitory potential might be beneficial as emergency care treatment for lethalness of Elapidae snake via inhibition of its component i.e AChE, present in very large amount with high catalytic activity (11). Plants extracts that hold alkaloids have been reported to possess anti-snake venom AChE activity (12).

The aim of this study was to evaluate the pharmacological effect of *Calligonum polygonoides* against Krait snake’s venom AChE and also to study the potential of *C. polygonoides* in treatment of Alzheimer’s disease.

## Materials and Methods

***Collection and processing of plant material***

*C. polygonoides* plant was collected from Township, Bannu district, KPK-Pakistan and was identified in department of Botany, UST Bannu. The voucher specimen was deposited at the herbarium of the department. It was cleaned, washed and shed dried at room temperature for three successive weeks. 200 g of milled powder of the plant was extracted in 1 L commercial grade methanol (Merck Lab) and was randomly shaken for 3 hr on a shaker machine and kept for 7 days at room temperature. After 7 days, the extract was sieved and the filtrate was further concentrated on rotary evaporator at 38 ^o^C under reduced pressure. The rotary evaporator product then applied to lyophilizer and converted into very fine powder form, which was stored at 4 ^o^C in falcon tube. About 5 mg/5 ml stock solution was prepared and was further diluted into different sub-solutions i.e. 100 µg/ml, 200 µg/ml, and 300 µg/ml for various pharmacological assays.

***Venom***

Venom from 10 live krait snakes (*Bungarus sindanus*) was bled manually and freeze dried immediately. It was stored at -20 ^o^C for further use. 

**Enzyme inhibition activity**

***Acetylcholinesterase assay***

The standard method of (13) was used with some alterations made by (14) for the cholinesterase activity assay. Rates of hydrolysis (V) were measured at different concentrations of the AChE (S) ranging from 0.5 to 1 mM, in 1 ml assay solution using 50 mM phosphate buffer pH 7.4 and 10 mM DTNB (5,5’-dithiobis-(2-nitrobenzoic acid). About 20 µl diluted snake venom was also added and the reaction mixture was incubated for 5 min at 37 ^o^C. Stock solution of plant extract was prepared as 1 mg/ml in methanol. The enzyme-substrate reaction immediately started upon the addition of different concentration of substrate. The hydrolysis was scrutinized by the formation of thiolate di-anion of DTNB every 15 sec during 90 sec using a spectrophotometer. The amount of yellow color due to formation of the thiolate dianion of DTNB at 412 nm was measured as an activity of AChE.

***Protein Estimation***

Using bovine serum albumin as standard, protein was assayed by the standard procedure of Bradford (1976) (15).

***Kinetic Studies***

 The kinetic study of interaction between* C. polygonoides* plant extract and AChE was measured using the Lineweaver Burk double reciprocal plot over a concentration of acetylthiocholine ranging from 0.5 to 1 mM in the presence and absence of extract. It was performed to estimate the type of inhibition. The substrate was added after incubating the reaction mixture for 5 min at 37^ o^C. The K_m_ and V_max_ values were obtained by plotting 1/V vs 1/S.

**Table 1:** Effect of methanolic extract of *Calligonum polygonoides* on Km (mM) and Vmax of krait snake venom acetylcholinesterase

Methanolic Extract of *Calligonum polygnoids* (µM)	K_m_ (mM)	% Increase	V_max_(µg AcSCh/min/mg protein)	% Decrease
0 100 200 300	0.16 0.22 0.26 0.33	0 25 62.5 106.6	1052 889 753 517	0 15.49 28.2 50.5

**Figure 1:**
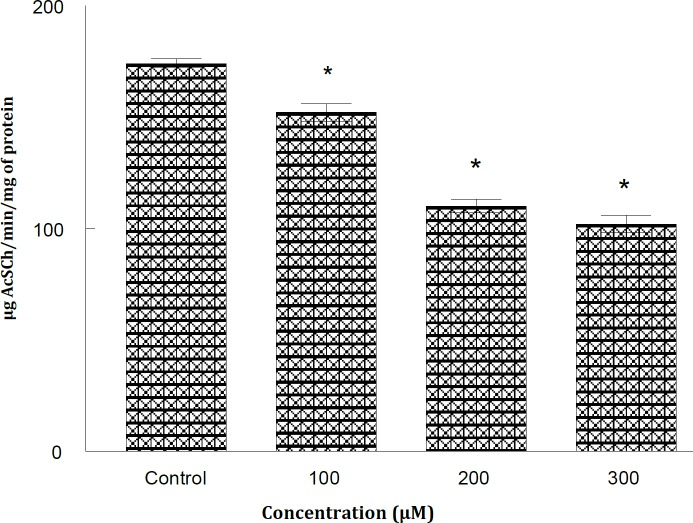
Concentration dependent inhibition of krait snake venom acetylcholinesterase in the absence and presence of methanolic extract of *Calligonum polygonoides* after 10 min incubation at 37 ^o^C. All experiments were repeated at least three times and similar results were obtained. * *P*<0.05. Significantly different from control

**Figure 2:**
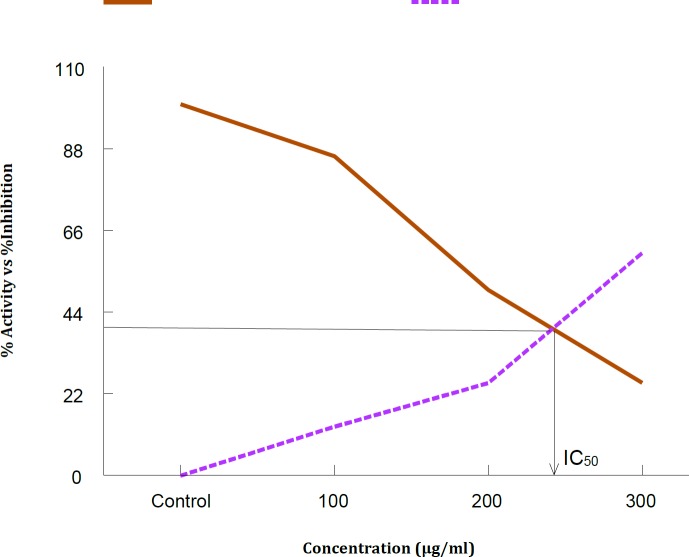
A plot of the percentage residual activity in the absence and presence of various concentration of *Calligonum polygonoides *methanolic extract after 10 min incubation at 37 ^o^C. About 0.5 mM acetylcholinesterase (AChE) was used as a substrate for snake venom AChE. The results represent the mean of three different experiments performed in duplicate

**Figure 3:**
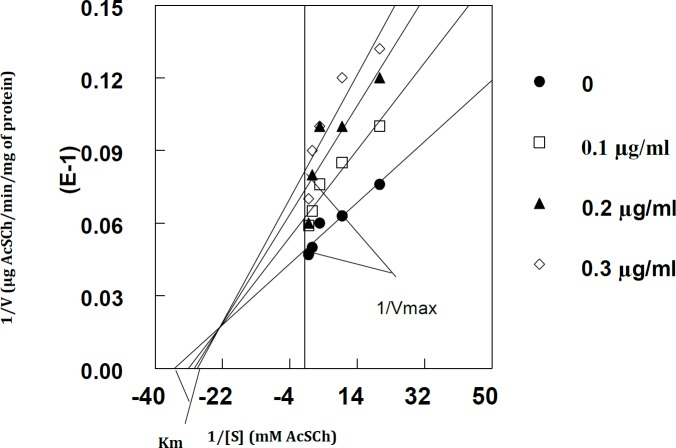
Methanolic extract of *Calligonum polygonoides* caused mixed type of inhibition of krait snake venom AcSCh. Data is expressed in the form of Lineweaver–Burk (reciprocal of enzyme velocity versus reciprocal of AcSCh) plot. The results represent the mean of three different experiments performed in duplicate by using different concentration of extract as shown in the legend boxes

## Results

*C. polygonoides* extract is effective against krait venom AChE since it could inhibit the venom AChE. The results of our finding also showed that the methanolic extract of *C. polygonoides* (100-300 µg/ml) altered the activity of krait snake venom AChE in a dose dependent manner (Table 1). The kinetic behavior of venom AChE was studied by increasing concentration of the extract (Figure 1). The Lineweaver-Burk plot and double reciprocal plot showed that the extract causes mixed type of inhibition in which the K_m_ value increased while 

V_max_ decreased (Figure 3) with increasing concentration of *C. polygonoides*. The IC_50_ value that was calculated by drawing simple graph of % activity of enzyme vs % inhibition of enzyme was 250 µg/ml (Figure 2). The increase in K_m_ value and decrease in V_max_ by increasing the concentration of *C. polygonoides* extract are shown in Table 1.

## Discussion

Snake bite is the major concern of developing countries, since the great majority of deaths occur due to envenomation. Anti-snake venom remedy is the only solution to the danger. Usually, horse sera are used to produce the anti-snake venom; however, horse sera contain immunoglobulins that results in side effects, which are compliment-mediated. Phyto-medication (plant based treatment) is known to be in use for long time (16) and numbers of plants are suggested for snake bite treatment (10). Krait snake venom contain large amount of AChE than any other source (17). In snake venom, it is in monomeric form while in all other source it is present in multimeric form (11). Therefore, snake venom AChE is the best source of drug design for the treatment of Alzheimer’s disease, which is due to deficiency of neurotransmitter acetylcholine. The hydrolysis of acetylcholine results in the formation of acetyl group and choline after it is transported to the synapses (18). Nowadays, the main strategy to enhance the amount of acetylcholine in Alzheimer’s patient is to inhibit or block the function of AChE. AChE inhibition is the major mode of action of most Alzheimer drugs.

The methanolic extract of *C. polygonoides* inhibits krait venom AChE in a dose dependent manner. Kinetic study indicated that the extract caused mixed type of inhibition. In this case, the K_m_ values increased and V_max_ decreased by increasing concentration of methanolic extract of *C. polygonoides*. In such type of inhibition, the substrate competes with inhibitor and sometimes binds with other side of the enzyme and as a result changes the conformation of enzyme resulting in decreased the catalytic activity. Such type of inhibition is reversible, and sufficient substrate molecules can overcome the inhibition. The results support one of the popular uses of species of the genus Zanthoxylum as anti-snake venom (12). The calculated IC_50 _is 250 µg/ml, which is less as compared to other tested plants extract indicating that the compounds present in the methanolic extract of *C. polygonoides* have potent AChE inhibitory activity. 

## Conclusion

*C. polygonoides *is a good source of natural compounds to block or inhibit the activity of AChE in krait snake venom. Moreover, this plant can be used as a therapeutic agent to cure Alzheimer’s disease due to inhibition of AChE activity, which causes enhancement in the amount of neurotransmitter (acetylcholine) in patient. 
